# Right Atrial Deformation Using Cardiovascular Magnetic Resonance Myocardial Feature Tracking Compared with Two-Dimensional Speckle Tracking Echocardiography in Healthy Volunteers

**DOI:** 10.1038/s41598-020-62105-9

**Published:** 2020-03-23

**Authors:** Vien T. Truong, Cassady Palmer, Michael Young, Sarah Wolking, Tam N. M. Ngo, Brandy Sheets, Chelsey Hausfeld, Allison Ornella, Michael D. Taylor, Karolina M. Zareba, Subha V. Raman, Wojciech Mazur

**Affiliations:** 1 The Christ Hospital Health Network, The Lindner Research Center, Cincinnati, Ohio USA; 2 The Sue and Bill Butler Research Fellow, The Lindner Research Center, Cincinnati, Ohio, USA; 3 Cincinnati Children’s Hospital Medical Center, Cincinnati, Ohio USA; 4 Ohio State University Wexner Medical Center, Columbus, Ohio USA; 5 Indiana University School of Medicine, Indianapolis, Indiana USA

**Keywords:** Cardiology, Medical imaging

## Abstract

Speckle tracking echocardiography (STE), and more recently, cardiovascular magnetic resonance myocardial feature tracking (CMR-FT) provides insight into all phases of atrial function. The aim of our study was to compare all phases of RA strain using CMR-FT and STE and also assess the relationship between RA and LA strain. A total of 61 healthy volunteers with mean age of 45 ± 13 years had adequate tracking for analysis on CMR-FT and 2D-STE. Females had larger RA reservoir strain (39 ± 15% vs. 32 ± 13%, p = 0.046) and conduit strain (26 ± 12% vs. 20 ± 9%, p = 0.03) when compared to males, but was not the case with booster strain (14 ± 7% vs. 12 ± 6%, p = 0.45). In comparison with STE derived strain, the RA reservoir and conduit strain were not significantly different between CMR-FT and the three echocardiography gating methods (p > 0.05 for all). Noticeably, there were no significant differences in strain and strain rate between RA and LA function using CMR-FT (p > 0.05 for all). RA strain and strain rate using CMR-FT had fair and good intra- and inter-observer reproducibility and had superior reproducibility compared to STE derived strain.

## Introduction

Right atrial (RA) strain is emerging as a promising technique for robust assessment of RA function^1,2^. For decades, the right heart has been underplayed in its contribution to overall cardiac function and has been affectionately coined the “forgotten heart”. While the right heart is no longer forgotten, it remains poorly understood and to date calls for further investigation. Speckle tracking echocardiography (STE), and more recently, cardiovascular magnetic resonance myocardial feature tracking (CMR-FT) provides insight into all phases of atrial function including reservoir, conduit, and booster^3–5^. Recently, RA dyssynchrony can be analysed by determining the time to peak strain in the reservoir phase or during atrial contractile phase^6^. RA function can be challenging by STE given the anatomic location of the RA, thin atrial wall, RA appendage, and the presence of superior and inferior vena cava^7,8^. On the contrary, given its higher spatial resolution and ability to define endocardial borders, CMR has long been accepted as the gold standard modality for assessment of the heart^9^. CMR-derived myocardial feature tracking (FT) is a technique analogous to echocardiography speckle tracking, deriving quantitative deformation parameters from routinely available steady state free precession (SSFP) cine sequence, and therefore does not require additional tagging sequence acquisitions^10^. While there are obvious advantages of CMR, RA strain using CMR compared with those using STE is currently lacking. The aim of our study was (1) compare all phases of RA strain using CMR-FT and STE, and (2) assess the relationship between RA and LA strain.

## Results

### Population characteristics

**Figure 1:**
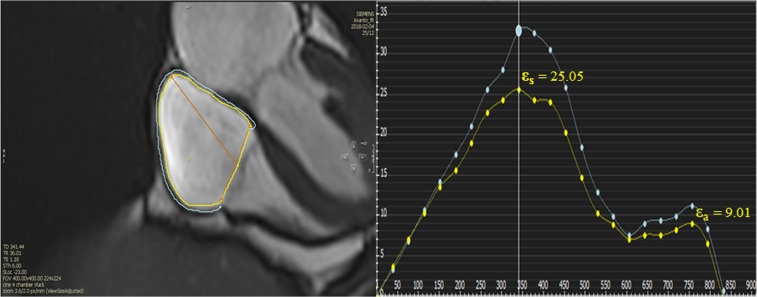
RA measurements by CMR- feature tracking in healthy volunteer. The yellow line is endocardial curve, the blue line is epicardial curve. ε_S_, reservoir strain; ε_a_, booster function. Global endocardial LA strain and strain rate values (yelllow line) were recorded.

A total of 61 healthy subjects were included in the study in proportions. Overall, the mean age of healthy subjects was 45 ± 13, of those, 31 (51%) were female. Baseline characteristics and volumetric chamber indices for all healthy subjects with gender difference are summarized in Table 1. Right ventricular ejection fraction were higher in females when compared to males (58 ± 6% vs 54 ± 7%, p = 0.01). In contrast, no gender difference in maximal and minimal RA volume were found (p = 0.32; p = 0.12, respectively). There was no correlation between RA strain and maximal and minimal RA volume, body surface area, body mass index, heart rate and RVEF (P > 0.05 for all) (Supplementary Table S1). Females had larger RA reservoir strain values (39 ± 15% vs. 32 ± 13%, p = 0.046) and conduit strain values (26 ± 12% vs. 20 ± 9%, p = 0.03) when compared to males (Table 2, Fig. 1). In regards to the RA booster strain no gender differences were found (14 ± 7% vs. 12 ± 6%, p = 0.45).

**Table 1:** Baseline characteristics for all healthy subjects and stratified according to gender.

Variable	All (n = 61)	Female (n = 31)	Male (n = 30)	P-Value (gender)
Age (years)	45 ± 13	44 ± 14	46 ± 13	0.65
BSA (m^2^)	1.93 ± 0.21	1.80 ± 0.16	2.07 ± 0.18	<0.001
BMI (kg/m^2^)	25.8 ± 2.9	25.4 ± 3.2	26.2 ± 2.7	0.32
SBP (mmHg)	125 ± 15	121 ± 14	128 ± 16	0.08
DBP (mmHg)	75 ± 11	73 ± 11	78 ± 11	0.07
Heart rate (bpm)	62 ± 9	63 ± 10	61 ± 9	0.41
Maximal RA volume	49 ± 16	47 ± 16	51 ± 16	0.32
Minimal RA volume	26 ± 9	24 ± 9	28 ± 9	0.12
RVEDVI (ml/m^2^)	65 ± 13	62 ± 14	69 ± 12	0.04
RVESVI (ml/m^2^)	29 ± 7	26 ± 7	32 ± 7	0.001
RVSVI (ml/m^2^)	36 ± 9	36 ± 9	37 ± 9	0.59
RVEF (%)	56 ± 7	58 ± 6	54 ± 7	0.01
LVEF (%)	61 ± 7	62 ± 6	60 ± 8	0.34

Normally distributed continuous variables are presented as mean ± standard deviation. Categorical variables presented as n (%).

BSA, body surface area; BMI, body mass index; SBP, systolic blood pressure; DBP, diastolic blood pressure; LVEDVI, Left ventricular end-diastolic volume index; RVESVI, Right ventricular end-systolic volume index; RVSVI, Right ventricular stroke volume index; RVEF, Right ventricular ejection fraction; LVEF, Left ventricular ejection fraction.

**Table 2:** Right atrial function stratified according to gender.

	All (n = 61)	Female (n = 31)	Male (n = 30)	P-Value (gender)
**Strain (%)**				
Reservoir	35.53 ± 14.35	39.21 ± 15.22	31.85 ± 12.61	0.046
Conduit	22.57 ± 11.08	25.62 ± 12.01	19.52 ± 9.29	0.03
Booster	12.96 ± 6.36	13.59 ± 7.05	12.33 ± 5.65	0.45
**Strain rate (s**^**−1**^**)**				
Reservoir	1.6 (1.3–2.2)	1.8 (1.3 to 2.5)	1.5 (1.2 to 2.2)	0.22
Conduit	−1.7 (−2.4 to −1.3)	−2.1 (−3.1 to −1.3)	−1.5 (−1.9 to −1.2)	0.03
Booster	−1.5 (−2.1 to −1.1)	− 1.7 (−2.4 to −1.1)	−1.5 (−2.0 to −1.1)	0.31

Normally distributed continuous variables are presented as mean ± standard deviation. Categorical variables presented as n (%).

### Comparison of RA Strain Using CMR-FT and 2D-STE and difference between LA and RA deformation using CMR-FT

In comparison with STE derived strain, the RA reservoir and conduit values were not significantly different between CMR-FT and the three echocardiography gating methods (p > 0.05 for all, Table 3). In regards to booster strain, the volume gating STE had a larger value compared to CMR-FT (16.17 ± 5.40% vs 12.96 ± 6.36%, p = 0.045), but not between CMR-FT and R-R gating STE (11.69 ± 4.23% vs 12.96 ± 6.36, p = 0.09) and CMR-FT and P-P gating STE (−13.69 ± 4.11% vs. 12.96 ± 6.36%, p = 0.96). Noticeably, there were no significant differences in strain and strain rate between RA and LA function using CMR-FT (p > 0.05 for all, Table 4).

**Table 3:** Comparison of RA strain using Cardiac Magnetic Resonance-feature tracking and speckle tracking echocardiography.

	CMR-FT	R-R gating	P-P gating	Volume gating	P^a^	P^b^	P^c^
**Strain**							
Reservoir	35.53 ± 14.35	35.92 ± 6.38	33.86 ± 6.23	39.66 ± 8.78	0.26	0.72	0.26
Conduit	22.57 ± 11.08	24.21 ± 7.15	20.16 ± 7.22	23.49 ± 8.67	0.81	0.64	0.15
Booster	12.96 ± 6.36	11.69 ± 4.23	−13.69 ± 4.11	16.17 ± 5.40	0.045	0.09	0.96

Normally distributed continuous variables are presented as mean ± standard deviation. Categorical variables presented as n (%).

^a^CMR vs. Volume gating.

^b^CMR vs. R-R gating.

^c^CMR vs. P-P gating.

*^*^Absolute value was used for comparison.

**Table 4:** Comparison of RA and LA function using CMR-FT.

Strain	RA function	LA function	P value
**Strain (%)**			
Reservoir	35.53 ± 14.35	36.60 ± 9.30	0.94
Conduit	22.57 ± 11.08	23.97 ± 8.33	0.85
Booster	12.96 ± 6.36	12.63 ± 4.49	0.61
**Strain rate (s**^**−1**^**)**			
Reservoir	1.6 (1.3 to 2.2)	1.81 (1.41 to 2.11)	0.98
Conduit	−1.7 (−2.4 to −1.3)	−2.01 (−2.61 to −1.51)	0.13
Booster	−1.5 (−2.1 to −1.1)	−1.81 (−2.31 to −1.31)	0.51

Normally distributed continuous variables are presented as mean ± standard deviation. Categorical variables presented as n (%).

### Intra- and inter-observer reproducibility

Intra- and inter-observer variability of RA strain, strain rate using CMR-FT were shown (Supplementary Table S2). There was fair and good intra- and inter-observer reproducibility. The RA strain had higher reproducibility as compared to the strain rate. The RA reservoir and conduit functions are more reproducible as compared to the booster function (Supplementary Table 2). The reproducibility of CMR-FT was superior when compared to STE derived strain.

## Discussion

The routine application of RA strain using CMR-FT in clinical practice requires knowledge of feasibility and reproducibility. To the best of our knowledge, this is the first study to compare all three phases of RA strain using CMR-FT with speckle tracking echocardiography, and also assess the relationship between RA and LA strain.

Assessment of RA size includes the measurement of RA diameters, area, and volume which has been accepted as a prognostic marker of mortality and outcomes^11–13^. However, RA volume does not encompass the entire spectrum of all phases of RA function. Additionally, measurement of right atrial volume using 2D echocardiography may be limited by angle dependency and geometric assumptions. Right atrial deformation using STE and CMR-FT have been used to identify the entire scope of RA function (reservoir, conduit, and booster function) and detect subclinical dysfunction^14^. Much work is being done to bridge the gap in understanding of how RA function plays into pulmonary artery hypertension, heart failure, congenital heart disease. Querejeta Roca *et al*. showed that pulmonary artery hypertension was associated with impaired RA reservoir and conduit function, independent of RA size and pressure, and likely reflects right ventricular dysfunction^2^. Hope *et al*. found that RA strain is potentially a useful prognostic tool for identifying PAH patients who are at risk for future adverse outcomes associated with RV failure^15^. In the setting of heart failure, changes in phasic function of the RA in response to changes in RV compliance has been noted and used to stratify patients at higher risk for complications^16^. RA dyssynchrony, detected using RA strain, was an independent predictor of paroxysmal atrial fibrillation in patients with atrial septal defects undergoing percutaneous closure and was a more sensitive parameter than left atrial indices in prediction of the following intervention^6^. Additionally, combined assessment of RA dyssynchrony and 3D-RA expansion index has been shown to be a robust marker for predicting paroxysmal atrial fibrillation and more sensitive than conventional volumetric atrial indices^6^.

Although STE has been the preferred modality for assessing RA function, CMR can overcome some of the limitations of STE and may be the superior modality for assessment of RA function without limitations by acoustic windows. Recent studies proved the feasibility of using CMR-FT for different pathophysiologic states. Dick *et al*. found patients with acute myocarditis had a decreased RA reservoir and conduit function by CMR-FT and can improve the diagnostic performance in patients with suspected myocarditis^17^. Kutty *et al*. demonstrated right longitudinal atrial strain using CMR-FT is feasible in patient with Tetralogy of Fallot. This study exhibited there was significant association of RA strain with RV dysfunction, independent of RA size and may serve as an useful parameter for assessing RV decompensation and prognosis^18^.

Our study showed a difference between gender composition and RA assessment of reservoir and conduit strain. Females had a larger magnitude in reservoir and conduit strain when compared to males, but was not the case with booster strain. This was concordant with previous studies using STE^14^ as well as using CMR^19^. Maceira *et al*. assessed RA function using volumetric method where RA reservoir, conduit, and booster function were calculated from maximum, minimum, and pre atrial contraction RA volume^19^. Of note, the volumetric method is an indirect method using volume to assess three phases of atrial function, while myocardial deformation using STE and CMR FT is a direct and novel technique for assessing the atrial function. Our study also demonstrated that there was no correlation found between RA strain and maximal and minimal RA volume which is different from LA data^20,21^. This may be explained by the presence of other structures (vena cava, appendage) which may interfere with the mechanics of the RA. Compared to previous studies, our result demonstrated a difference in normal RA strain values based on modality selection. Liu *et al*. demonstrated lower FT derived peak RA longitudinal strain values when compared to our current study^3^. Furthermore, their study did not provide the normal reference value for RA booster and conduit function. Contrastingly, Leng *et al*. reported RA reservoir, conduit, and booster values (53.9 ± 7.8%, 33.7 ± 8.2%, and 20.2 ± 5.6%) in a healthy group^4^, which were higher than our study. The difference in the contrasting values may be explained by different software and methods used to analyze strain (CVI 4.2 Tissue Tracking software in our study compared to QS strain software in aforementioned study). Additionally, Leng et. al did not report normal values of RA strain stratified according to gender. A notable limitation of these studies was the lack of direct comparison between all three phases of RA strain using both CMR-FT and STE.

Our analysis proved there was no significant difference in RA reservoir and conduit function using CMR-FT and STE. There was only a marginal P value (P = 0.045) for booster strain using volume gating STE which was larger when compared to CMR-FT. This finding may suggest that CMR-FT and STE can be used interchangeably in clinical practice. Our study also provided an insight into the interaction between RA and LA function, with no differences found between LA and RA strain using CMR-FT. In regard to the reproducibility, RA strain using CMR-FT had a good intra-observer and inter-observer reproducibility. Of note, reservoir and conduit strain had higher reproducibility than booster strain which is in agreement with previous study using STE^14^. Additionally, RA strain was more feasible and reproducible compared to strain rate which is similar to the previous study assessing LA strain using CMR-FT^5^. Importantly, RA strain using CMR-FT appeared potentially to be more accurate than 2D-STE given its superior reproducibility which may be explained by higher spatial resolution and ability to define endocardial borders by CMR.

### Limitations

This study had several limitations. Firstly, this study was a single-center study with a relatively smaller sample size. However, all healthy volunteers were recruited carefully and underwent CMR and comprehensive transthoracic echocardiogram within one hour of each other. Secondly, we did not perform direct comparisons with other techniques such as SENC or DENSE, as those sequences were not available at the time of examination. However, these techniques are time-consuming and challenging due to the very thin RA wall. Thirdly, RA strain was measured using an RV-specific software because no software was dedicated for RA analysis at the time of this study. Fourthly, myocardial deformation measurements using STE are known to vary among vendors and this may be true with CMR-FT. Finally, we only assessed global longitudinal strain and did not assess radial strain. Radial strain has already been noted for its difficulty to be obtained with poor reproducibility.

## Conclusion

Our data provides evidence that a comprehensive assessment of three phases of RA function including reservoir, conduit and booster strain with acceptable intra- and inter-observer reliability is feasible by CMR FT. In addition, RA strain assessment using CMR-FT with automated software makes analysis more time-efficient and practical. RA strain using CMR-FT and STE has good agreement and suggests that it may be used interchangeably in clinical practice.

## Methods

A total of 61 healthy volunteers were enrolled prospectively in this single-center study. The study was conducted under the approval of the Institutional Review Board of The Christ Hospital, Cincinnati, OH. Informed consent was obtained from each volunteer, and all procedures were performed according to the Declaration of Helsinki. The volunteer’s eligibility was determined by absence of cardiovascular risk factors (i.e. smoking, diabetes, and hypertension) as well as normal physical cardiac examination, electrocardiogram, and normal echocardiographic findings (defined as normal systolic and diastolic function with no more than mild valvar regurgitation). Exclusion criteria included known ventricular and supraventricular arrhythmia, obstructive sleep apnea, coronary artery disease, renal disease, BMI > 30.

Blood pressure and heart rate were recorded for each volunteer prior image acquisition for both the echocardiogram and sequence of CMR scans. Each volunteer underwent a cardiac magnetic resonance study and comprehensive transthoracic echocardiogram within one hour of each other.

### CMR

Subjects underwent cine CMR on a 1.5T Siemens scanner at Christ Hospital, using standardized imaging protocol. True FISP sequences with breath-hold were performed to obtain CMR acquisitions comprising a stack of contiguous parallel short axis slices covering the entire LA, LV, RA and RV from base to apex, a stack of contiguous parallel two and four chamber long axis slices covering the left ventricle, and one LV long axis slice 3 chamber acquisition. The slice thickness/spacing was 8 mm/1.6 mm for short axis imaging, and 6 mm/1.2 mm for long axis imaging.

### CMR image post-processing

Volumetric analysis was performed with commercially available software (Circle Cardiovascular Imaging, Calgary, Canada) in a random order by a blinded experienced observer. Minimal, maximal, right atrial volumes were calculated at the respective cardiac phase using short axis cine images in the 4-chamber views excluding inferior, superior vena cava and the RA appendage. RV end-diastolic volume (RVEDV), RV end-systolic volume (RVESV), ejection fraction (EF) were measured using Circle CVI 4.2, with applicable values indexed to body surface area then calculated.

### CMR feature tracking analysis

**Figure 2:**
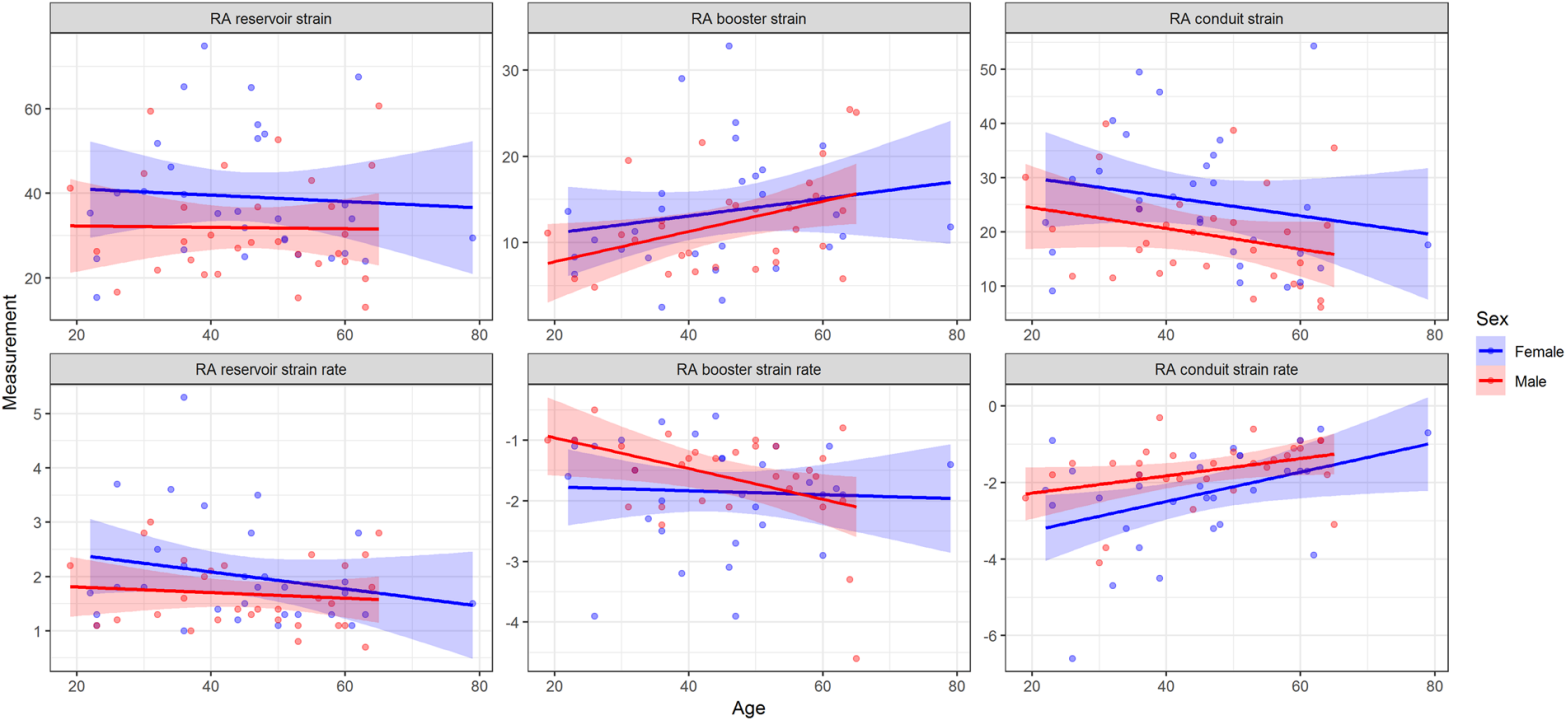
Normal RA function stratified according to gender and age. RA booster function gradually increased with age, with a decrease in RA conduit function in order to preserve reservoir function.

The RA myocardial deformation was quantified using CVI 4.2 Tissue Tracking software (Circle Cardiovascular Imaging, Calgary, Canada). The software then constructed a deformable myocardial model based on the tracing, assuming the myocardium was nearly incompressible. In each of the subsequent frames, the displacement of the myocardial tissue, including the borders, were automatically determined using a gradient-based optical flow method with an incompressible model constraint. The propagated myocardial tissue across the cardiac cycle was verified by the operator to ensure the accuracy of propagation. At end-diastole, endocardial and epicardial borders were manually delineated using a point-and-click approach before the automated tracking algorithm was applied. Atrial endocardial and epicardial border contours were initially traced in the apical four chamber views at end-diastole. Endocardial atrial strain values for each tissue point as well as the global strain values were automatically derived by the software (Fig. 2, Supplementary Video). Feature-tracking techniques for the assessment of atrial phasic strain has been previously described^5,22^.

### Speckle tracking echocardiography analysis

A comprehensive 2D transthoracic echocardiogram was performed using an EPIQ7 system, with an X5-1 phased array probe (Philips Medical Systems, Andover, MA). Each volunteer was scanned in the left lateral position. ECG leads were placed on the volunteer’s chest with clear delineation of the QRS complex, T wave, and P wave being prerequisite to image capture. Care was taken to optimize all 2D images by employing breath holds, adjustments to overall gain, time gain compensation, and compression controls. Image acquisition for all strain images were obtained with breath holds then optimized for visualization of both ventricular and atrial endocardial borders. Strain image acquisition was obtained using a consecutive three beat capture and recorded in a digital raw data format. Optimized atrial acquisition was obtained by foreshortening ventricular anatomy. All acquisitions were stored digitally and analyzed off line.

All strain analysis was performed off line by one experienced sonographer with 15 years of experience. Dedicated atrial strain software (Tom Tec Imaging Systems) was used to analyze atrial strain. Atrial strain was performed utilizing apical-four chamber views. A manual point–and-click method for tracing was used along the compacted atrial myocardium. Care was exercised to exclude inferior, superior vena cava, pulmonary veins and the atrial appendage. Tom Tec uses a 3 segment ROI for each apical view for atrial analysis. Studies were excluded if two or more segments could not be tracked secondary to discontinuation of atrial tissue (drop out) or suboptimal image quality. The zero-baseline time reference for initiation of atrial strain curves were based on three different gating methods (Supplementary Figure). The same endocardial contours were used for consistency of all three methods. Ventricular end-diastole was automatically detected by the software and noted to occur within the QRS complex. This represented the start of the R-to-R interval used for the R-R gating method. These values reflected a positive reservoir value because the atrium expands during ventricular systole. Atrial strain curves were also initiated based on the zero-time reference set to atrial contraction (baseline zero set to the onset of the P wave). This determined the P-P values obtained for analysis^23^. Finally, atrial strain curves were initiated from establishing baseline zero as the minimal atrial volume obtained from a simultaneously generated volume curve. Peak strain values were obtained at the same point where the peak volume was identified.

### Reproducibility

Inter-observer variability was assessed in 20 random healthy subjects by two investigators (V.T. and M.Y.). The same images were analyzed by each operator, who saved the results independently of the other, to provide a blinded assessment. The intra-observer measurements were performed on average 1 month apart or longer in a random order to avoid recall bias.

### Statistical analysis

Categorical data are presented as frequency (percentage) and comparison between groups was performed using the chi-square test or Fisher exact test. Continuous variables are presented as mean ± standard deviation (SD) for normal or expressed as median (interquartile range) for non-normal distribution. Differences in continuous variables between two groups were analyzed using Student’s t-test or the Mann-Whitney U test as appropriate. Comparisons between three or more groups were made with a one-way analysis of variance (ANOVA). Difference between two methods (CMR-FT and STE) were tested by paired sample t-test. Intra- and inter-observer reproducibility for RA strain, strain rate parameters were analyzed using the Bland–Altman method^24^ and intra-class correlation coefficients (ICC) (two-way random, absolute agreement). Agreement was considered excellent when ICC > 0.74, good when ICC = 0.60–0.74, fair when ICC = 0.40–0.59, and poor when ICC < 0.4^25^. Pearson correlation was used to analyze the relationships between RA parameters with heart rate, BSA, blood pressure, and right ventricular ejection fraction. A two-sided P-value of <0.05 was considered statistically significant. The statistical analyses were performed using IBM SPSS Statistics for Windows, version 22.0 (IBM Corp., Armonk, N.Y., USA) and R software, version 3.5.3 (The R Foundation, Vienna, Austria)^26^.

### Ethics approval and consent to participate

The study was approved by the institutional review board of the Christ Hospital Health Network. All volunteers provided written informed consent for participation in this study.

## Supplementary information

Supplementary Figure.

Supplementary Tables.

Supplementary Video.

Supplementary Information.

## Data Availability

In order to protect participant confidentiality, data are available from the The Christ Hospital Institutional Data Access/Ethics Committee for researchers who meet the criteria for access to confidential data. Requests for data access can be sent to the corresponding author at wojciech.mazur@thechristhospital.com.
