# Supplementary figures and images for: Right Atrial Deformation Using Cardiovascular Magnetic Resonance Myocardial Feature Tracking Compared with Two-Dimensional Speckle Tracking Echocardiography in Healthy Volunteers
Source: Sci Rep. 2020 Mar 23;10:5237. doi: 10.1038/s41598-020-62105-9 (PMC7089993; doi:10.1038/s41598-020-62105-9)

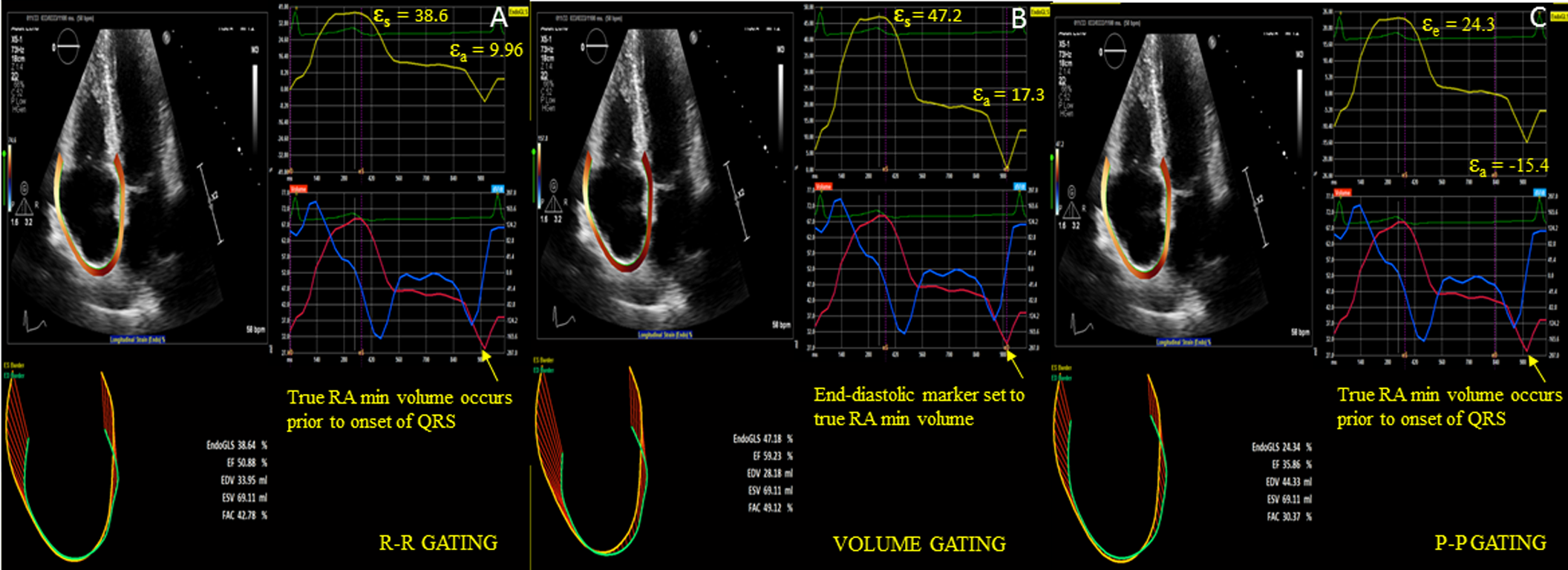

Supplement: Supplementary file 1 — Supplementary Figure. [file 41598_2020_62105_MOESM1_ESM.tif]
