# Supplementary material for: Right Atrial Deformation Using Cardiovascular Magnetic Resonance Myocardial Feature Tracking Compared with Two-Dimensional Speckle Tracking Echocardiography in Healthy Volunteers
Source: Sci Rep. 2020 Mar 23;10:5237. doi: 10.1038/s41598-020-62105-9 (PMC7089993; doi:10.1038/s41598-020-62105-9)
Supplement: Supplementary file 2 — Supplementary Tables. [file 41598_2020_62105_MOESM2_ESM.docx]

**Right Atrial Deformation Using Cardiovascular Magnetic Resonance Myocardial Feature Tracking Compared with Two-Dimensional Speckle Tracking Echocardiography in Healthy Volunteers**

Vien T. Truong^1,2^, MD; Cassady Palmer^1^, BS; Michael Young^1^, AAS; Sarah Wolking^1^, AAS; Tam NM. Ngo^1^, MD; Brandy Sheets^1^, BS; Chelsey Hausfeld ^1^, BS; Allison Ornella^1^, AAS, Michael Taylor^3^, MD; Karolina M. Zareba^4^, MD; Subha V. Raman^4^, MD; Wojciech Mazur^1*^, MD

**Supplementary Table S1**. Correlation between RA strain and baseline characteristics

|  | Maximal RA volume | | Minimal RA volume | | BSA | | BMI | | Heart Rate | | RVEDV | | RVESV | | RVEF | |
| --- | --- | --- | --- | --- | --- | --- | --- | --- | --- | --- | --- | --- | --- | --- | --- | --- |
| **RA Strain** | r | P | r | P | r | P | r | P | r | P | r | P | r | P | r | P |
| Reservoir | 0.05 | 0.72 | -0.11 | 0.40 | -0.24 | 0.06 | 0.06 | 0.65 | -0.09 | 0.51 | -0.07 | 0.57 | -0.13 | 0.31 | 0.13 | 0.34 |
| Conduit | 0.13 | 0.32 | -0.04 | 0.75 | -0.22 | 0.09 | 0.09 | 0.51 | -0.15 | 0.26 | -0.02 | 0.86 | -0.10 | 0.46 | 0.14 | 0.27 |
| Booster | -0.12 | 0.35 | -0.17 | 0.18 | -0.17 | 0.20 | -0.02 | 0.89 | -0.02 | 0.88 | -0.13 | 0.32 | -0.13 | 0.32 | 0.03 | 0.79 |
| r, Pearson Correlation, BSA, body surface area; BMI, body mass index; RVESV, Right ventricular end-systolic volume; RVSV, Right ventricular stroke volume; RVEF, Right ventricular ejection fraction; | | | | | | | | | | | | | | | | |

**Supplementary Table S2.** Intra- and inter-observer reproducibility of right atrial function

|  | **Intra-observer variability** | | | | **Inter-observer variability** | | | |
| --- | --- | --- | --- | --- | --- | --- | --- | --- |
|  | Mean difference ± SD | 95% CI | P value | ICC | Mean difference | 95% CI | P value | ICC |
| **CMR** |  |  |  |  |  |  |  |  |
| **Strain (%)** |  |  |  |  |  |  |  |  |
| Reservoir | 0.04 ± 10.01 | -4.65 to 4.73 | 0.99 | 0.84 | 0.23 ± 8.26 | -4.09 to 3.64 | 0.90 | 0.89 |
| Conduit | 0.01 ± 6.38 | -2.97 to 2.99 | 0.99 | 0.91 | 0.02 ± 6.13 | -2.85 to 2.88 | 0.99 | 0.91 |
| Booster | 0.03 ± 6.04 | -2.80 to 2.86 | 0.98 | 0.63 | -0.24 ± 3.74 | -1.99 to 1.51 | 0.78 | 0.74 |
| **Strain rate** |  |  |  |  |  |  |  |  |
| Reservoir | 0.12 ± 0.80 | -0.25 to 0.49 | 0.51 | 0.56 | 0.08 ± 0.85 | -0.32 to 0.48 | 0.68 | 0.52 |
| Conduit | 0.20 ± 0.84 | -0.19 to 0.59 | 0.31 | 0.85 | 0.16 ± 0.84 | -0.24 to 0.55 | 0.42 | 0.84 |
| Booster | -0.03 ± 0.85 | -0.43 to 0.37 | 0.88 | 0.67 | -0.08 ± 0.62 | -0.37 to 0.22 | 0.59 | 0.81 |
| **STE** |  |  |  |  |  |  |  |  |
| **Strain (%)** |  |  |  |  |  |  |  |  |
| Reservoir | -0.51 ± 6.36 | -3.48 to 2.46 | 0.72 | 0.64 | -5.64 ± 5.97 | -8.43 to -2.84 | < 0.001 | 0.55 |
| Conduit | -0.60 ± 5.32 | -3.08 to 1.89 | 0.62 | 0.83 | -5.68 ± 7.34 | -9.11 to -2.24 | < 0.001 | 0.58 |
| Booster | 0.09 ± 3.56 | -1.58 to 1.75 | 0.92 | 0.88 | 0.04 ± 4.60 | -2.11 to 2.19 | 0.97 | 0.71 |
| **Strain rate** |  |  |  |  |  |  |  |  |
| Reservoir | 0.02 ± 0.28 | -0.11 to 0.14 | 0.81 | 0.44 | -0.10 ± 0.22 | -0.20 to -0.003 | 0.045 | 0.58 |
| Conduit | 0.07 ± 0.33 | -0.08 to 0.22 | 0.35 | 0.37 | 0.22 ± 0.30 | 0.07 to 0.36 | 0.005 | 0.47 |
| Booster | -0.09 ± 0.25 | -0.20 to 0.03 | 0.15 | 0.74 | -0.12 ± 0.28 | -0.25 to 0.01 | 0.07 | 0.59 |
| Normally distributed continuous variables are presented as mean ± standard deviation. Categorical variables presented as n (%)  95% CI, Confidence Interval | | | | | | | | |
