# Supplementary material for: Right Atrial Deformation Using Cardiovascular Magnetic Resonance Myocardial Feature Tracking Compared with Two-Dimensional Speckle Tracking Echocardiography in Healthy Volunteers
Source: Sci Rep. 2020 Mar 23;10:5237. doi: 10.1038/s41598-020-62105-9 (PMC7089993; doi:10.1038/s41598-020-62105-9)
Supplement: Supplementary file 4 — Supplementary Information. [file 41598_2020_62105_MOESM4_ESM.docx]

**Supplementary Figure**. RA strain using speckle tracking echocardiography (4 chamber view). (A and C). R-R gating and P-P gating, true minimum left atrial volume occurs prior to initiation of the QRS complex. B. Volume gating, End-diastolic marker (baseline zero) is set to true minimal right atrial volume for initiation of strain analysis. Top graph: strain curve, Bottom graph: volume curve. ε_S_, Reservoir strain, ε_e_, and conduit strain, ε_a_, booster function.

**Supplementary video**. Right atrium longitudinal strain in the 4-chamber views
